# Radiomics machine learning models for lung cancer early diagnosis in heterogenous multicentre chest CT data: LIBRA study results

**DOI:** 10.1186/s41747-026-00791-2

**Published:** 2026-10-05

**Authors:** Benjamin Hunter, Prashanthi Ratnakumar, Daniel Tong, Nishanthi Senthivel, Amyn Bhamani, Abigail Coe, Susannah Bloch, Samuel V. Kemp, Simon Doran, Neal Navani, Arjun Nair, Jonathan Ratoff, Claire E. Wells, Justin Garner, Andrew Nicolson, Catey Bunce, Matthew Blackledge, Anand Devaraj, Eric O. Aboagye, Richard W. Lee

**Affiliations:** 1 Imperial College London, London, England; 2 The Royal Marsden NHS Foundation Trust, London, England; 3 Imperial College Healthcare Trust, London, England; 4 University College London Hospitals NHS Foundation Trust, London, England; 5 Epsom and St Helier University Hospitals NHS Trust, Surrey, England; 6 Nottingham University Hospitals NHS Foundation Trust, Nottingham, England; 7 The Institute of Cancer Research, London, England; 8 The Royal Brompton and Harefield Hospitals, Guy’s and St Thomas’s NHS Foundation Trust, London, England

**Keywords:** Early detection of cancer, Lung neoplasms, Machine learning, Radiomics, Tomography (x-ray computed)

## Abstract

**Objective:**

We developed and validated radiomics models for lung cancer prediction in indeterminate pulmonary nodules that retain performance in heterogenous data from multiple centres.

**Materials and methods:**

The retrospective Lung Imaging Biobank for Radiomics and AI (LIBRA) study recruited 1,026 patients (2,071 computed tomography scans) with 5-to-30-mm lung nodules from seven UK hospitals (01/07/2020–30/09/2022). Benign and malignant nodules were segmented for feature extraction using PyRadiomics. Principal component analysis reduced feature dimensionality to twenty components. Logistic regression models using ComBat correction, fixed effects or random effects to mitigate for disparate data across study centres were compared. Models were fit to the whole dataset and evaluated using 10-fold cross-validation. Internal-external validation assessed between-centre heterogeneity. Models were benchmarked against volume, Brock score and scrambled voxels. Calibration was assessed using calibration curves. Decision-curve analysis was used to assess net benefit.

**Results:**

Between-centre discrimination variability was observed with an area under the curve (AUC) ranging 0.75–0.91. The mixed-effects model provided better discrimination over ComBat or fixed effects alone (AUC 0.91 (95% confidence interval [CI]: 0.90–0.93) *versus* 0.75 (95% CI: 0.72–0.78) and 0.88 (95% CI: 0.86–0.90), respectively (*p* < 0.0001 and 0.003, respectively) in cross-validation. Radiomics features retained significance in multivariable models including established clinical features. The final model was superior to models developed using volume alone, Brock score, and scrambled-voxels. The model was well calibrated (intercept -0.036, slope 0.89 with net benefit compared to volume or Brock score alone.

**Conclusion:**

Radiomics models show between-centre heterogeneity, with resilience best achieved with mixed-effects models. The final model had high performance with net benefit over established risk models.

**Key Points:**

***Question*** Can heterogeneous data from multiple centres be used to predict lung nodule malignancy risk?

***Findings*** Radiomics cancer prediction models must account for heterogeneity to maintain performance between different centres. The mixed-effects model achieved AUC 0.91 (cross-validation) for cancer prediction.

***Relevance statement*** The model provided net benefit for nodule investigation over Brock score and volume alone and could assist in decision-making following prospective validation.

**Graphical Abstract:**

**Figure:**
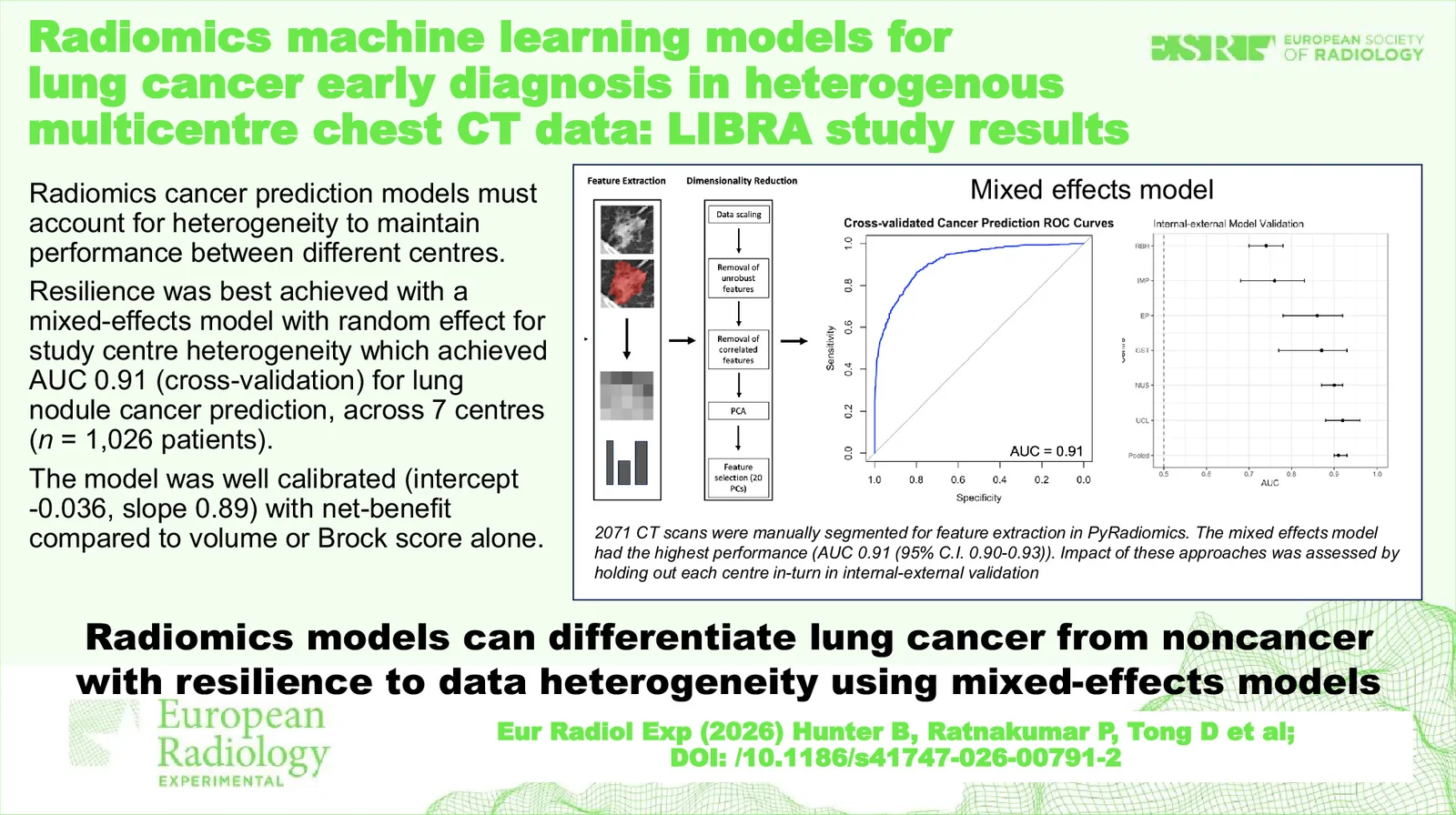

## Background

The use of diagnostic computed tomography (CT) is increasing globally [1]. So too is the detection of incidental pulmonary nodules, with some 1.6 million people diagnosed annually in the USA alone [2]. As most nodules are benign, correctly finding the small proportion (~3–5%) of early-stage lung cancers is challenging [2–4]. Although guidelines exist to stratify nodules using risk scores such as that developed by Brock et al [5], reliance on serial scans generates uncertainty for patients and is resource intensive. Particularly in light of workforce concerns following the COVID-19 pandemic, methods to improve nodule risk-stratification are needed [6].

In recent years, many deep learning (DL) models have been developed to assist nodule detection and stratification [7, 8]. In 2019, Ardila et al at Google used data from the USA National Lung Cancer Screening Trial to develop a convolutional neural network (CNN)-based algorithm for classification [9]. The model achieved a high-discrimination capability with an area under the receiver operating characteristic curve (AUC) of 0.94 and improved on radiologist predictions. Similarly, Optellum’s ‘Lung Cancer Prediction CNN’, which was also developed using the National Lung Cancer Screening Trial data, has been validated in three UK centres with high performance, with an AUC of 0.90 [10]. While DL models have achieved promising results, they require large datasets to train and validate, and their inner workings can be difficult to explain [11]. Jacobs and Ginneken suggest that the ‘black box’ nature of such algorithms may limit their acceptability to patients and raise concerns that proprietary code protected by commercial licensing could limit scientific transparency and reproducibility [12].

Hand-crafted radiomics models are attractive because they are more readily explainable, and features can be extracted using open-source libraries, improving transparency and data sharing [13]. Much work has been done by the Imaging Biomarker Standardisation Initiative–IBSI to allow calibration of feature extraction programmes across institutions to improve reproducibility [14]. To this end, many hand-crafted radiomics models have been recently developed for nodule stratification [15–20].

However, few have been developed using heterogeneous, clinically-utilised computed tomography (CT) images from multiple centres, and while some have been externally validated, the impact of study centre on performance is often overlooked [21, 22]. It is also unclear how best to account for centre-specific effects on radiomics features [23, 24]. With this in mind, the Lung Imaging Biobank for Radiomics and AI (LIBRA) study was established with the following aims: (1) to develop lung cancer prediction models using heterogeneous data from multiple centres; (2) to assess how model performance may vary between centres, and how accounting for this variation impacts performance; (3) to perform robust benchmarking against clinical risk factors, and (4) to assess the ‘net benefit’ of the final radiomics model for clinical decision support.

## Materials and methods

### Ethics approval

Health Regulatory Authority–HRA and research ethics committee–REC approval were obtained for this retrospective cohort study (IRAS ID: 274775, REC reference 20/NI/0088, ClinicalTrials.gov: NCT04270799). Patient consent was not required. Data from 502 of the recruited patients were previously used to develop a large lung nodule cancer prediction model [22].

### Study sample

**Fig. 1:**
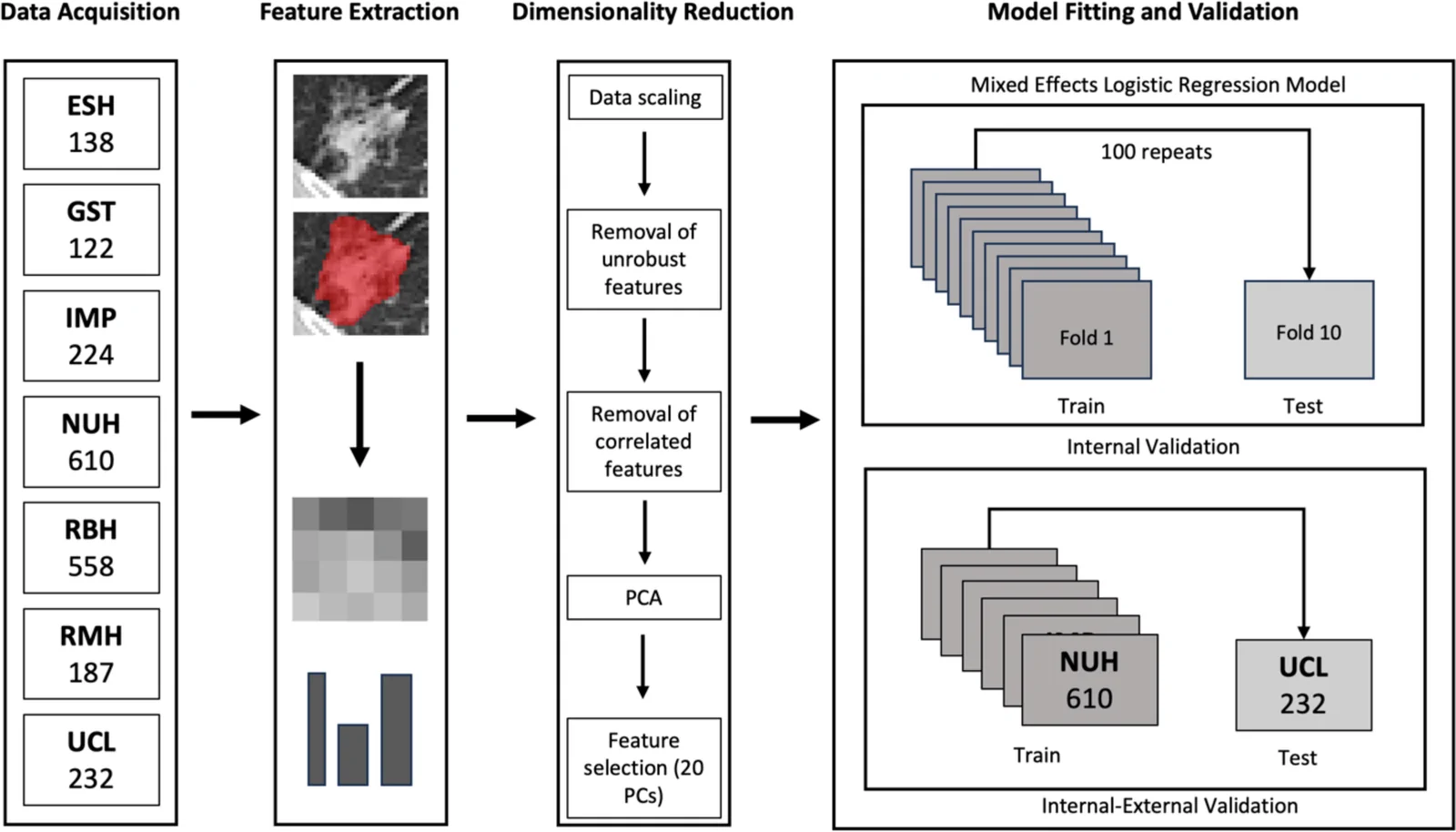
Study recruitment and feature processing. A total of 2,071 CT scans were manually segmented for feature extraction in PyRadiomics v3.1.0. Features with low test–retest and inter-reader reliability (intraclass correlation coefficient < 0.8) and with high correlation (*r* > 0.95) were removed before principal component analysis. The components accounting for 100% of the data variation (20 PCs) were retained for model fitting. The mixed-effects logistic regression model was validated using 10-fold cross-validation, and an internal-external validation approach. The final model was fit to the whole dataset. ESH, Epsom and St. Helier; GST Guy’s and St. Thomas’; IMP Imperial College London Hospitals; NUS, Nottingham University

Patients were recruited between 1st July 2020 and 30th September 2022 by the clinical teams at the seven participating centres (Fig. 1). Eligible patients were aged over 18 with 5–30-mm indeterminate pulmonary nodules on baseline CT imaging. ‘Indeterminate’ refers to nodules not detected via screening with an unclear aetiology at the outset. Criteria for benignity were matched to the British Thoracic Society guidelines [25]. Benign nodules had to be either shrinking, stable for one year on volumetry or stable for two years using transaxial diameter. Benign subsolid nodules required stability for four years. All malignant nodules were biopsy-proven. Scans over 2.5 mm thickness or nodules with unknown ground truth were excluded. Acquisition parameters are included in Supplementary Table S1. Up to three CT scans (baseline, interim and the final follow-up) were included for each patient. Patient scans were grouped by fold identifier to prevent leakage when splitting. The final diagnosis at the last scan defined the ground truth for all other scans for each patient. Only a single nodule per scan was included. Five hundred and two patients from the LIBRA study were previously used to develop a large-nodule-specific model [22]. Clinical data were obtained using electronic healthcare record review by their parent teams. Data were link-anonymised and saved to the study data collection tool (Excel, version 16.93.1, Microsoft, Redmond, Washington, USA) before upload to LIBRA-XNAT.

### Image processing and feature extraction

Digital Imaging and Communication in Medicine (DICOM) images were anonymised at participating centres using a custom DICOM Browser script, before upload to the study Extensible Neuroimaging Archive Toolkit (XNAT) server. Corresponding clinical data were link-anonymised before upload. No imputation was used for missing data. DICOM Images were converted to Neuroimaging Informatics Technology Initiative–NIfTI format using dcm2niix (https://github.com/rordenlab/dcm2niix). Manual segmentation was performed by a consultant clinical oncologist with 9 years’ experience (BH) using ITK-Snap (v4.0, https://www.itksnap.org/pmwiki/pmwiki.php). Image and mask voxels were resampled to 1 mm^3^ using BSpline and nearest-neighbour interpolation, respectively. Features were extracted using PyRadiomics (v3.1, Harvard and Dana-Farber Cancer Institute) with a binwidth of 25 HU and otherwise default parameters.

A total of 107 features were extracted from first-order, grey level co-occurrence matrix—GLCM, grey level dependence matrix—GLDM, grey level run length matrix—GLRLM, grey level size zone matrix—GLSZM, neighbouring grey tone difference matrix—NGTDM, and shape categories. Features were scaled using *Z*-standardisation. The reference image database to evaluate response to therapy in lung cancer–RIDER lung dataset, obtained from the cancer imaging archive, was used to assess feature robustness to longitudinal imaging (test–retest) and inter-reader segmentation variability (Supplementary Table S2) [23, 24, 26]. Forty-two features with intraclass correlation coefficients ≥ 0.8 for test–retest and inter-reader agreement were retained for model development [27].

### Statistical analysis

Statistical oversight was provided by statisticians at The Royal Marsden Hospital (RMH) and bioinformaticians at the Institute of Cancer Research. All analyses were conducted in RStudio (v4.1.2, https://posit.co/downloads). A formal power calculation was not performed due to the exploratory nature of the work; thus, the recruitment target of 1,000 patients was based on expert consensus only. All statistical tests were two-sided, with statistical significance defined as *p* < 0.05. Model discrimination was evaluated using the AUC, with 95% confidence intervals (CIs) generated using the cvAUC R package based on the variance of out-of-fold predictions. The Brier score was used to measure accuracy of predictions, based on the mean squared difference between predicted probabilities and actual outcomes.

### Model development and validation

The first of each pair of highly correlated radiomics features (*r* ≥ 0.95) was removed before performing principal component analysis–PCA to reduce data dimensionality. The first 20 principal components (PCs) explained 100% of the data variation and were retained for model development. Correlations between PCs and original features were explored using Pearson’s correlation. As an external test set was not pre-specified, radiomics models were fit to the entire dataset and evaluated using ten-fold cross-validation. Fold identifiers were fixed to allow comparison between different models. Due to observed complete separation, fixed-effect models were fit using Firth’s logistic regression (R logistf package).

Three radiomics models were developed to determine malignancy *versus* benignity, which included a fixed effect for nodule density, and: (1) ComBat correction for study centre and contrast status; (2) fixed effects for contrast status and study centre; and (3) random effects for study centre and fixed effects for contrast status (mixed effects model). For internal-external validation, models were fit with one centre held out in turn (except for RMH, which included only malignant nodules), to estimate between-centre variability [28–31]. Out-of-sample cross-validation predictions were used to assess model discrimination and calibration. Calibration curves were generated by fitting a Locally Estimated Scatterplot Smoothing–LOESS smoother to predicted probabilities using the rms package, and net benefit graphs were generated using the R dca package. The highest performing radiomics model in internal cross-validation was selected as the final model. The final model was benchmarked against a combined model including established clinical risk factors, a volume-only model, a model based on the Brock score [5] and a model generated using scrambled voxels (the ‘null’ model).

The anonymised radiomics data used to generate the model used in this study have been deposited into the Mendeley Data repository, DOI: 10.17632/3xc6gtgcb3.1. R code to replicate model generation is available from the GitHub link: https://github.com/dr-benjamin-hunter-mtw/LIBRA. Anonymised clinical imaging data is available on reasonable request to the corresponding author, subject to institution data sharing procedures.

## Results

### Patient characteristics

The final study cohort consisted of 1,026 patients with 2,071 CT scans. The breakdown of clinicodemographic information by centre is shown in Table 1. Overall, 491 (48%) patients were male, and 456 (65% of those recorded) had a positive smoking history; 560 (55%) patients had benign nodules. While the proportion of male and female participants was well-matched amongst the centres, heterogeneity was observed for other demographic variables, including age, smoking status, nodule density, nodule ground truth, reconstruction kernels and the proportion of contrast-enhanced scans. Some of this heterogeneity is explained by the inclusion of a tertiary cancer centre (RMH), which recruited only malignant nodules from an older patient cohort (mean age 75.6 years), with a higher proportion of smokers (85%) and contrast-enhanced scans (61%).

**Table 1:** Patient demographic details

Variable	EP	GST	IMP	NUH	RBH	RMH	UCL
Scans	138	122	224	610	558	187	232
Age							
Mean (SD)	66.40 (13.24)	62.03 (10.81)	68.95 (10.82)	65.44 (11.86)	66.55 (10.27)	75.48 (8.61)	63.43 (13.07)
Sex							
Male (%)	20 (38)	28 (48)	63 (48)	112 (45)	170 (52)	56 (46)	42 (47)
Female (%)	33 (62)	30 (52)	68 (52)	135 (55)	156 (48)	66 (54)	47 (53)
Smoking							
Positive (%)	32 (60)	33 (57)	89 (70)	142 (58)	NR	102 (84)	58 (65)
Negative (%)	21 (40)	25 (43)	42 (30)	105 (42)	NR	20 (16)	31 (35)
Nodule density							
Solid (%)	49 (93)	48 (83)	99 (76)	230 (93)	223 (68)	97 (80)	57 (64)
SSN/GGO (%)	4 (7)	10 (17)	32 (24)	17 (7)	103 (32)	25 (18)	32 (36)
Nodule diagnosis							
Benign (%)	47 (89)	50 (86)	95 (73)	165 (67)	123 (38)	0 (0)	80 (90)
Malignant (%)	6 (11)	8 (14)	36 (27)	82 (33)	203 (62)	122 (100)	9 (10)
Contrast status							
Enhanced (%)	47 (35)	37 (30)	38 (17)	206 (30)	223 (40)	114 (61)	75 (32)
Non-contrast (%)	91 (65)	85 (70)	186 (83)	406 (70)	335 (60)	73 (39)	157 (68)
CT Kernel							
Sharp (%)	136 (99)	42 (34)	115 (51)	55 (9)	387 (69)	92 (49)	27 (12)
Soft (%)	2(1)	80 (66)	109 (49)	557 (91)	171 (31)	95 (51)	205 (88)

*GGO* Ground-glass opacity, *sd* Standard deviation, *SSN* Sub-solid nodule. Centres: *GST* Guy’s and St. Thomas’, *IMP* Imperial College London Healthcare Trust, *NUS* Nottingham University Hospitals, *RBH* Royal Brompton Hospital, *UCL* University College London Hospitals

### Principal component analysis

The cumulative proportion of variation explained by each of the radiomics PCs is shown in Table 2. The first five PCs explained 86% of the variance and were most correlated with GLCM_Idn, LDM_DependenceEntropy, GLSZM_SizeZoneNonUniformityNormalized, NGTDM_Busyness, and NGTDM_Contrast.

**Table 2:** Principal component analysis results

Feature	Variance	SD	Most correlated feature	*r*
PC1	0.45	3.75	original_glcm_Idm	0.89
PC2	0.66	2.65	original_gldm_DependenceEntropy	0.77
PC3	0.78	1.97	original_glszm_SizeZoneNonUniformityNormalized	0.85
PC4	0.83	1.25	original_ngtdm_Busyness	0.43
PC5	0.86	0.98	original_ngtdm_Contrast	0.35
PC6	0.89	0.91	original_firstorder_10Percentile	0.47
PC7	0.91	0.85	original_shape_Maximum3DDiameter	0.30
PC8	0.93	0.76	original_shape_Maximum2DDiameterSlice	0.32
PC9	0.94	0.68	original_glrlm_GrayLevelNonUniformity	0.31
PC10	0.95	0.60	original_glszm_SizeZoneNonUniformityNormalized	0.23
PC11	0.96	0.53	original_ngtdm_Contrast	0.33
PC12	0.97	0.50	original_shape_Maximum2DDiameterSlice	0.25
PC13	0.97	0.42	original_glrlm_GrayLevelNonUniformity	0.15
PC14	0.98	0.38	original_firstorder_10Percentile	0.22
PC15	0.98	0.35	original_gldm_GrayLevelNonUniformity	0.15
PC16	0.99	0.34	original_shape_Maximum2DDiameterColumn	0.16
PC17	0.99	0.33	original_shape_Maximum2DDiameterRow	0.18
PC18	0.99	0.29	original_shape_MinorAxisLength	0.18
PC19	0.99	0.26	original_shape_LeastAxisLength	0.17
PC20	1.00	0.24	original_glcm_Imc2	0.14

*Idm* Inverse difference moment, *Imc* Informational measure of correlation, *PC* Principal component, *sd* Standard deviation

### Centre effects

**Fig. 2:**
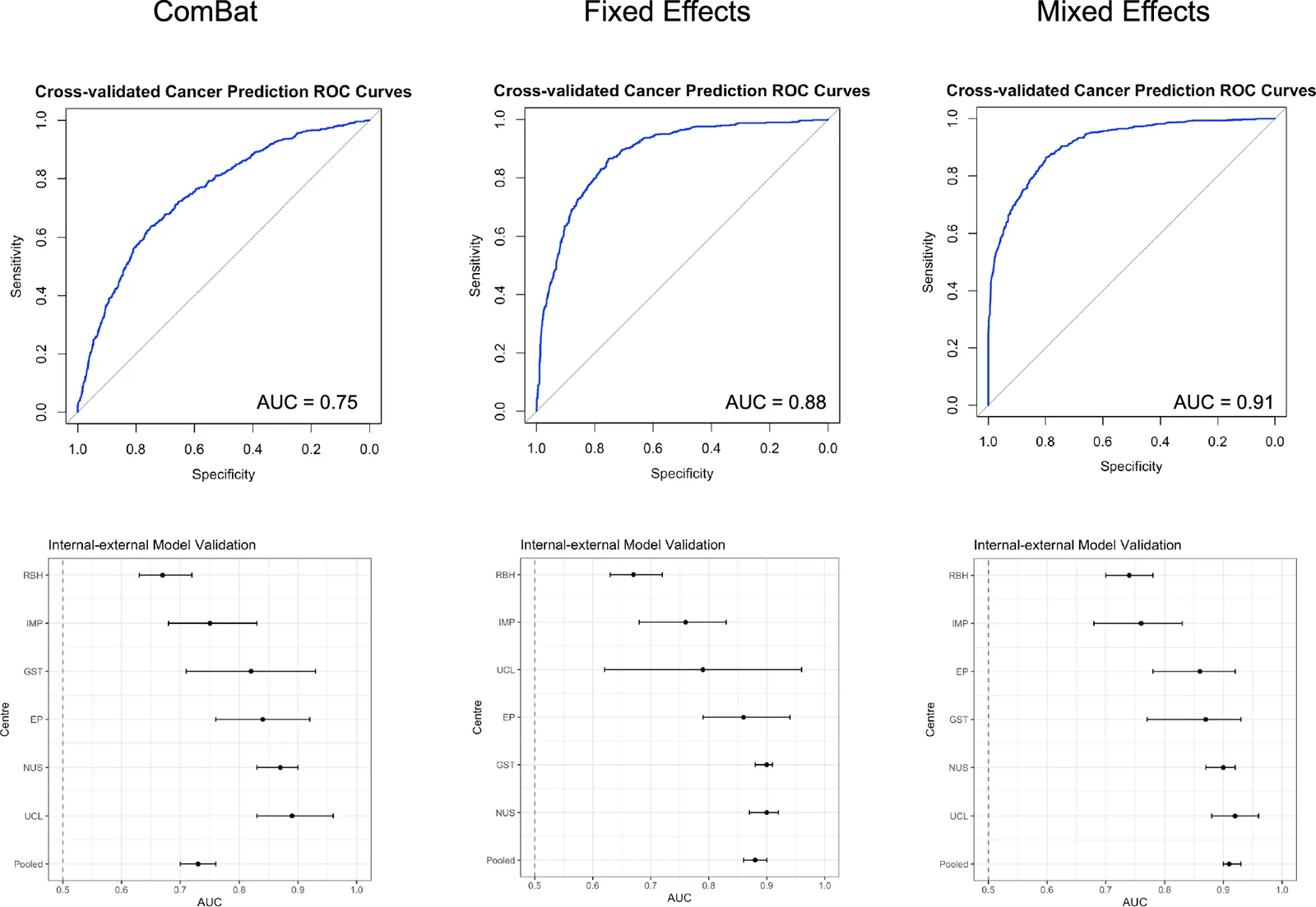
Model performance metrics. The out-of-sample predictions across 10-fold cross-validation were used to generate receiver operating characteristic curves (top row) for models utilising ComBat batch correction, fixed or random effects for study centre. The mixed effects model had the highest performance (area under the curve [AUC] 0.91, 95% CI: 0.90–0.93). The impact of these approaches was assessed by holding out each centre in turn in internal-external validation (bottom row). The ‘Pooled’ metrics represent internal cross-validation AUCs. GST, Guy’s and St. Thomas’; IMP, Imperial College London Healthcare Trust; NUS, Nottingham University Hospitals; RBH, Royal Brompton Hospital; UCL, University College London Hospitals

Three approaches were explored to mitigate between-centre heterogeneity in radiomics features (Fig. 2). Contrast status and nodule density were also included in these models, due to their likely effect on radiomics features. Firstly, ComBat batch correction was used to adjust for the effect of study centre and IV contrast. Secondly, a fixed-effect model including variables for study centre was developed. Because RMH included only malignant nodules, its inclusion as a fixed effect led to unstable coefficient estimates, and so these patients were removed from this model. Thirdly, a mixed effect model including random effects for study centre was fit. The ComBat model had the worst discrimination (AUC 0.75, 95% CI: 0.72–0.78). The inclusion of random effect terms led to statistically significant improvements in model discrimination over fixed effects alone (AUC 0.91 (95% CI: 0.90–0.93) *versus* 0.88 (95% CI: 0.86–0.90), *p* = 0.003), and was selected as the final model. Statistically significant improvements over COMBAT and fixed effects models were retained when RMH was excluded from all models (*p* < 0.0001 and 0.02, respectively). Model coefficients are shown in Table 3.

**Table 3:** Coefficients for the final mixed-effects model

Random effects			
Groups	Name	Variance	SD
Institution	Intercept	4.47	2.11

Fixed effects			
Variable	Beta	OR	*p*
PC1	0.64	1.90 (1.67–2.16)	< 0.0001
PC2	0.12	1.12 (0.99–1.28)	0.07
PC3	-0.47	0.63 (0.47–0.84)	0.001
PC4	-0.60	0.55 (0.44–0.69)	< 0.0001
PC5	-0.40	0.67 (0.37–1.21)	0.2
PC6	-0.42	0.66 (0.46–0.94)	0.02
PC7	-1.16	0.31 (0.19–0.51)	< 0.0001
PC8	-0.83	0.43 (0.24–0.78)	0.006
PC9	-1.07	0.34 (0.21–0.57)	< 0.0001
PC10	-0.64	0.53 (0.32–0.86)	0.01
PC11	-1.04	0.35 (0.12–1.06)	0.06
PC12	-0.55	0.58 (0.39–0.85)	0.006
PC13	-0.003	1.0 (0.45–2.23)	0.99
PC14	0.29	1.33 (0.66–2.67)	0.42
PC15	0.67	1.96 (0.87–4.41)	0.10
PC16	0.46	1.58 (1.04–2.4)	0.03
PC17	-0.68	0.51 (0.25–1.04)	0.06
PC18	-0.39	0.68 (0.39–1.18)	0.17
PC19	0.58	1.79 (0.98–3.25)	0.06
PC20	0.86	2.34 (1.08–5.04)	0.03
Contrast	0.51	1.66 (1.25–2.20)	0.0005
Solid density	-0.13	0.87 (0.44–1.75)	0.70
Sub-solid density	0.66	1.94 (1.01–3.72)	0.045

The 20 PCs accounting for 100% of the variance were retained for model fitting

*OR* Odds ratio, *PC* Principal component, *SD* Standard deviation

### Model calibration and net benefit

**Fig. 3:**
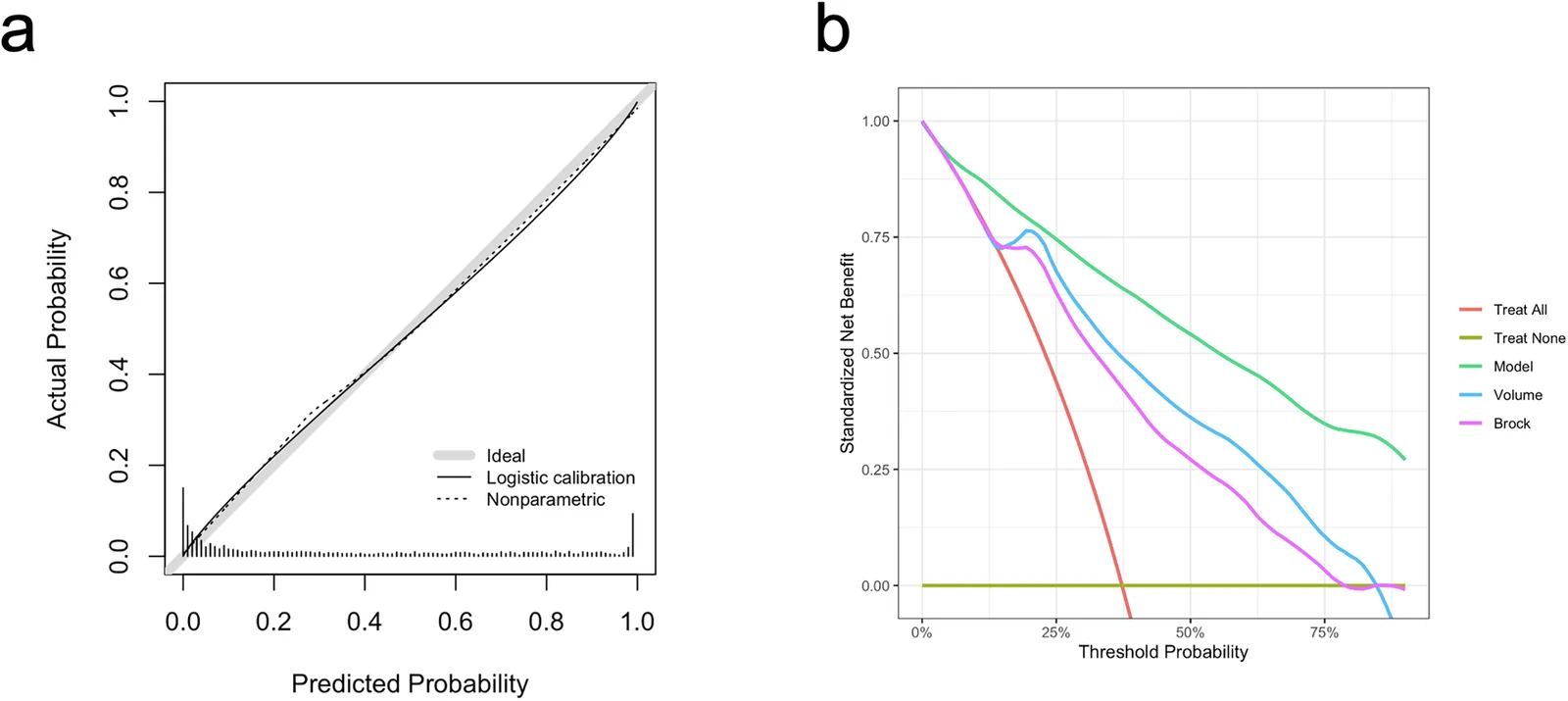
Final model calibration and net benefit. **a** Out-of-sample predictions across 10-fold cross-validation were used to fit a calibration slope using a Locally Estimated Scatterplot Smoothing smoother. The model was informative (Brier 0.11) and had good calibration (intercept -0.036, slope 0.89). **b** The net benefit of investigating patients based on cancer predictions from the radiomics model was compared to volume alone (blue), the Brock score (pink), investigating all (red) or investigating none (yellow). The separation of the curves indicates the model provides benefit over existing clinical decision tools

The final radiomics model calibration and net benefit graphs are shown in Fig. 3. The model was informative (Brier score 0.11, non-informative Brier threshold = 0.23) and well calibrated (intercept -0.036, slope 0.89). The model provided net benefit over cancer prediction models based on volume alone, the Brock score alone, and the ‘investigate all’ or ‘investigate none’ scenarios (Fig. 3b). At the 10% and 20% risk thresholds, the net benefit of investigating based on the model was 0.33 and 0.30, respectively, compared to 0.30 and 0.27 for the Brock score. However, using these thresholds for classification was suboptimal, with no malignant nodules in the model in 10% and 20% risk groups (accuracy 63%, sensitivity 0%, specificity 100%, PPV N/A and NPV 63%). At threshold probabilities over 25%, the standardised net benefit curves improved (Fig. 3b). Using a probability threshold which maximised the Youden index (0.3186) in out-of-fold predictions, example classification metrics were as follows: accuracy: 0.82 (95% CI: 0.81–0.84), sensitivity 0.86, specificity 0.80, positive predictive value 0.72, and negative predictive value 0.91.

### Model benchmarking

To assess the contribution of texture features, the radiomics model was compared to volume-only and scrambled voxel models. The radiomics model had statistically significantly better discrimination than the volume-only and scrambled voxel models: AUC 0.91 (95% CI: 0.90–0.93) *versus* 0.87 (95% CI: 0.86–0.87) and 0.75 (95% CI: 0.74–0.75), respectively.

The coefficients for the clinical variable-only model are shown in Supplementary Table S3. Age, smoking status, nodule size, upper lobe location, spiculation, intravenous contrast, non-solid density, male sex and the presence of multiple nodules were significant risk factors. The clinical variable-only model AUC for cancer prediction was lower than the radiomics model (0.87, 95% CI: 0.86–0.89, *p* < 0.0001). Radiomics PCs retained significance when combined with clinical variables (Supplementary Table S4), and the ANOVA test indicated that adding the 20 radiomics PCs to the clinical features led to improvements in model fit (Akaike information criterion 1,336 *versus* 1,684, *p* < 0.0001). Inclusion of clinical variables improved the AUC to 0.92 (95% CI: 0.91–0.93, *p* < 0.0001).

## Discussion

Through the LIBRA study, clinically utilised CT scans were used to develop a radiomics model for lung cancer prediction, which addresses heterogeneity between study centres. The final model achieved good discrimination (AUC 0.91, 95% CI: 0.90–0.93) and was well calibrated (intercept -0.036, slope 0.89). Radiomics features added useful information to models based on clinical factors alone, and the final model showed net benefit over three-dimensional volume and the Brock score, which are currently used for nodule risk stratification.

The methods adopted within this work address several limitations in the existing literature and the major challenge of implementation of radiomics and artificial intelligence in clinical practice. Firstly, though other studies have reported good discrimination with conventional radiomics models, few have been developed using data from multiple centres [17, 18, 20, 32]. The internal-external validation approach allowed robust estimation of model performance between centres, while utilising the full breadth of the data for model fitting and avoiding the limitations of split-sample methods [29, 31]. Secondly, while “Least Absolute Shrinkage and Selection Operator”–LASSO has been widely adopted for feature selection, simulation studies suggest it can be unstable and may not identify the ‘true’ predictors reliably [33]. By including the radiomics PCs accounting for 100% of the variability, the risk of losing important predictors was kept to a minimum. Thirdly, mixed-effects regression was adopted to account for clustering within institutions. We found variable performance of the model according to study centre, which is consistent with the existing literature suggesting centre-specific acquisition parameters may impact radiomics features, though there is no consensus on how to best account for this [27].

Batch-effect correction using ComBat has been proposed, but criticisms have been raised about the risk of false positives [34]. In the present study, ComBat adjustment led to model degradation. By including mixed-effects terms, the current model estimates and accounts for sources of feature variability without these potential drawbacks of batch correction. Model stability was also improved by the exclusion of features with poor test–retest reproducibility in an independent data set [23]. Finally, in recent years the importance of safeguards to prove radiomics models are measuring texture as well as volume has been emphasised. In 2019, Welch et al showed that a head and neck cancer prognostic model was measuring a surrogate for three-dimensional volume with little contribution from texture features [35]. By comparing the full model to (1) the volume model, wherein three-dimensional volume was the only radiomics feature, and (2) the null model, wherein CT voxels were scrambled, the contribution of textural analysis to the model has been proven.

For the broader radiomics community, this work highlights that models require detailed evaluation of between-centre heterogeneity to discover and account for important variations in performance and efforts made to develop algorithms that do not lose performance when undergoing validation in data with high variability, typical outside of clinical trial settings. Such work may help drive the adoption of radiomics in the clinical setting that are resilient to heterogenous real-world data. The ultimate aim is for radiomics algorithms to integrate into the Picture Archiving and Communication System–PACS workflow and provide real-time decision support to radiologists by supplementing visual reads with texture-based malignancy risk estimations.

There are some limitations to consider. As shown in Table 1, heterogeneity for several clinical and radiological factors was observed, reflecting the broad range of participating centres. The cause of observed performance variability is not certain, but may be related to nodule size, as the two worst-performing centres, RBH and Imperial, had on average much larger diameter nodules (20.5 ± 4.86 *versus* 13.7 ± 6.59, mean ± standard deviation). This is consistent with the previous hypothesis that models may perform better if stratified by size and requires further investigation [22]. This will be further evaluated using external validation studies stratified by size. It is also important to consider that the lack of a predefined external test set could increase the risk of model overfitting.

The final model fit to all data showed promising performance, but further external validation is warranted. It is also important to note that models based on volume or clinical risk factors alone achieved excellent discrimination AUCs of 0.87 (95% CI: 0.86–0.89) and 0.87 (95% CI: 0.86–0.87). The radiomics model was significantly superior, and showed net benefit in decision curve analysis, but whether the modest improvement in AUC to 0.92 (95% CI: 0.91–0.93) in the final model with clinical data included, translates into a clinically meaningful benefit requires further evaluation. At the 10 and 20% risk thresholds, the model did not classify malignancy well, as all patients had benign nodules in these cohorts. For the model to improve clinical stratification, further work combining model predictions with Brock thresholds, or classification using cutoff values derived from this study rather than the BTS algorithm, would be required. Finally, the model has been robustly benchmarked against existing risk factors such as volume and the Brock score, but how it may assist human readers in nodule classification has not been investigated. Reader studies with radiologists are an area for future development. The other area for future development is integration into clinical pipelines. At present, the model depends on manual segmentation, DICOM conversion to Neuroimaging Informatics Technology Initiative format, feature extraction using Python and model predictions in RStudio. Work to unify this pipeline into a single streamlined process for the end user is ongoing. There are currently several commercial tools available for nodule segmentation, which may be able to integrate with this predictive model.

In summary, data from multiple centres has been pooled to create a model for lung cancer prediction, using open-source software and publicly available libraries, advancing understanding of mitigation for heterogeneity. This model, which maintained good performance across centres in internal-external validation, requires further validation in an independent data set.

## Supplementary information

**Additional file 1: Supplementary Table 1:** Scan Acquisition Parameters by Institution. **Supplementary Table 2:** Intra-class correlation coefficients (ICCs) for the 42 radiomics features retained from the RIDER lung study. **Supplementary Table 3:** Coefficients for the clinical variable only model. **Supplementary Table 4:** Coefficients for the combined clinical and radiomics model. **Supplementary Table 5:** Radiomics Quality Score.

## Data Availability

The anonymised radiomics data used to generate the models used in this study have been deposited into the Mendeley Data repository, https://doi.org/10.17632/3xc6gtgcb3.1. R code to replicate model generation is available from the GitHub link: https://github.com/dr-benjamin-hunter-mtw/LIBRA. Anonymised clinical imaging data is available on reasonable request to the corresponding author, subject to institutional data sharing procedures.

## Abbreviations

AUC: Area under the (receiver operating characteristic) curve
CI: Confidence interval
CT: Computed tomography
DICOM: Digital imaging and communication in medicine
LIBRA: Lung imaging biobank for radiomics and AI (study)
PC: Principal component
