# Supplementary material for: Radiomics machine learning models for lung cancer early diagnosis in heterogenous multicentre chest CT data: LIBRA study results
Source: Eur Radiol Exp. 2026 Oct 5;10:149. doi: 10.1186/s41747-026-00791-2 (PMC13638909; doi:10.1186/s41747-026-00791-2)
Supplement: Supplementary file 1 [file 41747_2026_791_MOESM1_ESM.pdf]

Radiomics machine learning models for lung cancer early diagnosis in heterogenous multicentre chest CT data: LIBRA study results

ELECTRONIC SUPPLEMENTARY MATERIAL

Institution	Vendor	Kernel	n
EP	GE MEDICAL SYSTEMS	BONEPLUS	54
EP	GE MEDICAL SYSTEMS	CHST	9
EP	GE MEDICAL SYSTEMS	LUNG	47
EP	GE MEDICAL SYSTEMS	STANDARD	2
EP	SIEMENS	['Br60f', '3']	4
EP	TOSHIBA	FC55	22
GST	GE MEDICAL SYSTEMS	STANDARD	2
GST	Philips	B	38
GST	Philips	C	21
GST	Philips	CB	1
GST	Philips	D	4
GST	Philips	L	7
GST	Philips	YA	1
GST	Philips	YC	12
GST	SIEMENS	B70f	1
GST	SIEMENS	Br36d	1
GST	SIEMENS	['Bl57d', '3']	1
GST	SIEMENS	['Bl64d', '3']	15
GST	SIEMENS	['Br40d', '3']	10
GST	SIEMENS	['I31f', '3']	7
GST	TOSHIBA	FC08	1
IMP	GE MEDICAL SYSTEMS	LUNG	3
IMP	GE MEDICAL SYSTEMS	STANDARD	4
IMP	Philips	B	43
IMP	Philips	C	1
IMP	Philips	L	44
IMP	Philips	YA	16
IMP	Philips	YB	25
IMP	Philips	YC	1
IMP	SIEMENS	B20f	1
IMP	SIEMENS	B30f	11
IMP	SIEMENS	B70f	5
IMP	SIEMENS	['I26f', '3']	3
IMP	SIEMENS	['I30f', '2']	1
IMP	SIEMENS	['I30f', '3']	35
IMP	SIEMENS	['I31f', '3']	10
IMP	SIEMENS	['I70f', '3']	21
NUS	Canon Medical Systems	BODY_SHARP	2
NUS	GE MEDICAL SYSTEMS	CHST	3
NUS	GE MEDICAL SYSTEMS	LUNG	4
NUS	GE MEDICAL SYSTEMS	SOFT	5
NUS	GE MEDICAL SYSTEMS	STANDARD	87

NUS	Philips	B	109
NUS	Philips	C	4
NUS	Philips	L	3
NUS	SIEMENS	B30s	1
NUS	SIEMENS	B31f	1
NUS	SIEMENS	B35f	2
NUS	SIEMENS	B65f	2
NUS	SIEMENS	['Br40f', '3']	43
NUS	SIEMENS	['Br64f', '3']	2
NUS	SIEMENS	['I26f', '3']	2
NUS	SIEMENS	['I26f', '4']	2
NUS	SIEMENS	['I30f', '4']	5
NUS	SIEMENS	['I30f', '5']	3
NUS	SIEMENS	['I31f', '2']	2
NUS	SIEMENS	['I31f', '3']	4
NUS	SIEMENS	['I40f', '2']	14
NUS	SIEMENS	['I40f', '3']	155
NUS	SIEMENS	['I41f', '2']	9
NUS	SIEMENS	['I41f', '3']	97
NUS	SIEMENS	['I70f', '3']	29
NUS	SIEMENS	['I70f', '4']	1
NUS	TOSHIBA	FC07	10
NUS	TOSHIBA	FC08	9
NUS	TOSHIBA	FC18	2
RBH	GE MEDICAL SYSTEMS	BONEPLUS	6
RBH	GE MEDICAL SYSTEMS	CHST	5
RBH	GE MEDICAL SYSTEMS	DETAIL	1
RBH	GE MEDICAL SYSTEMS	LUNG	12
RBH	GE MEDICAL SYSTEMS	SOFT	3
RBH	GE MEDICAL SYSTEMS	STANDARD	29
RBH	GE MEDICAL SYSTEMS	ULTRA	1
RBH	Philips	B	3
RBH	Philips	C	3
RBH	Philips	E	1
RBH	Philips	L	3
RBH	Philips	YB	1
RBH	SIEMENS	B20f	16
RBH	SIEMENS	B20s	2
RBH	SIEMENS	B30f	42
RBH	SIEMENS	B31f	3
RBH	SIEMENS	B40f	9
RBH	SIEMENS	B45f	34
RBH	SIEMENS	B50f	15

RBH	SIEMENS	B60f	16
RBH	SIEMENS	B60s	2
RBH	SIEMENS	B70f	257
RBH	SIEMENS	B80f	1
RBH	SIEMENS	['I30f', '3']	1
RBH	SIEMENS	['I70f', '3']	34
RBH	TOSHIBA	FC03	16
RBH	TOSHIBA	FC10	6
RBH	TOSHIBA	FC12	1
RBH	TOSHIBA	FC13	2
RBH	TOSHIBA	FC30	19
RBH	TOSHIBA	FC51	8
RBH	TOSHIBA	FC52	2
RBH	TOSHIBA	FC83	1
RBH	TOSHIBA	FC86	3
RMH	GE MEDICAL SYSTEMS	BONEPLUS	14
RMH	GE MEDICAL SYSTEMS	CHST	22
RMH	GE MEDICAL SYSTEMS	LUNG	11
RMH	GE MEDICAL SYSTEMS	SOFT	10
RMH	GE MEDICAL SYSTEMS	STANDARD	28
RMH	Philips	B	5
RMH	Philips	YA	1
RMH	SIEMENS	B20f	33
RMH	SIEMENS	B30f	5
RMH	SIEMENS	B31f	4
RMH	SIEMENS	B60f	19
RMH	SIEMENS	B70f	6
RMH	SIEMENS	B75f	1
RMH	SIEMENS	D30f	1
RMH	SIEMENS	['I30f', '3']	1
RMH	SIEMENS	['I31f', '3']	3
RMH	SIEMENS	['I70f', '3']	13
RMH	TOSHIBA	FC07	3
RMH	TOSHIBA	FC08	4
RMH	TOSHIBA	FC08-H	1
RMH	TOSHIBA	FC12	1
RMH	TOSHIBA	FC85	1
UCL	GE MEDICAL SYSTEMS	LUNG	6
UCL	GE MEDICAL SYSTEMS	STANDARD	42
UCL	Philips	B	1
UCL	SIEMENS	B20f	61
UCL	SIEMENS	B26f	10
UCL	SIEMENS	B30f	1

UCL	SIEMENS	B31f	16
UCL	SIEMENS	B70f	6
UCL	SIEMENS	['Bf37f', '3']	14
UCL	SIEMENS	['I30f', '2']	7
UCL	SIEMENS	['I70f', '2']	3
UCL	TOSHIBA	FC03	2
UCL	TOSHIBA	FC07	8
UCL	TOSHIBA	FC07-H	34
UCL	TOSHIBA	FC08-H	8
UCL	TOSHIBA	FC18-H	1
UCL	TOSHIBA	FC86	12

Supplementary Table 1: Scan Acquisition Parameters by Institution.

Feature	Test-Retest ICC	Inter-Reader ICC
original_glcm_Imc1	0.96	0.96
original_shape_SurfaceVolumeRatio	0.93	0.97
original_glszm_GrayLevelNonUniformity	0.92	0.98
original_ngtdm_Contrast	0.92	0.96
original_glcm_Imc2	0.91	0.97
original_ngtdm_Strength	0.90	0.92
original_ngtdm_Coarseness	0.90	0.90
original_glrlm_RunLengthNonUniformity	0.89	0.98
original_ngtdm_Busyness	0.89	0.99
original_gldm_DependenceNonUniformity	0.89	0.97
original_shape_VoxelVolume	0.89	0.98
original_shape_MeshVolume	0.89	0.98
original_glcm_Idmn	0.88	0.98
original_glszm_SizeZoneNonUniformity	0.88	0.97
original_firstorder_RobustMeanAbsoluteDeviation	0.87	0.94
original_shape_MinorAxisLength	0.87	0.97
original_glrlm_GrayLevelNonUniformity	0.87	0.98
original_glszm_SmallAreaEmphasis	0.87	0.98
original_firstorder_MeanAbsoluteDeviation	0.86	0.93
original_firstorder_InterquartileRange	0.86	0.94
original_shape_Maximum2DDiameterColumn	0.86	0.97
original_glszm_SizeZoneNonUniformityNormalized	0.86	0.98
original_gldm_GrayLevelNonUniformity	0.86	0.99
original_glcm_Contrast	0.85	0.97
original_shape_LeastAxisLength	0.85	0.99
original_glcm_Idn	0.85	0.97
original_firstorder_Variance	0.84	0.91
original_gldm_GrayLevelVariance	0.84	0.91
original_glcm_DifferenceVariance	0.84	0.96
original_glrlm_GrayLevelVariance	0.84	0.91
original_shape_SurfaceArea	0.83	0.96
original_shape_Maximum2DDiameterRow	0.83	0.95
original_gldm_DependenceEntropy	0.83	0.90
original_glcm_DifferenceAverage	0.83	0.97
original_glszm_GrayLevelNonUniformityNormalized	0.82	0.92
original_gldm_SmallDependenceEmphasis	0.82	0.99
original_firstorder_10Percentile	0.82	0.94
original_shape_Maximum3DDiameter	0.82	0.95
original_gldm_DependenceNonUniformityNormalized	0.81	0.99
original_glszm_ZonePercentage	0.81	0.99
original_shape_Maximum2DDiameterSlice	0.81	0.95
original_glcm_SumSquares	0.80	0.89

Supplementary Table 2: Intra-class correlation coefficients (ICCs) for the 42 radiomics features retained from the RIDER lung study.

Random Effects:			
**Groups**	**Name**	**Variance**	**SD**
Institution	Intercept	5.95	2.44
**Fixed Effects:**			
**Variable**	**Beta**	**OR**	**p**
Age	0.01	0.02 (0-0.16)	0.02
FH	1.07	1.01 (1-1.03)	0.12
Smoking	1.07	2.91 (0.77-11.07)	< 0.0001
Size	0.08	2.91 (1.99-4.24)	< 0.0001
Upper Lobe	0.39	1.09 (1.06-1.12)	< 0.0001
Spiculation	1.54	1.48 (1.15-1.9)	< 0.0001
Contrast	0.87	4.68 (3.42-6.4)	< 0.0001
GGO Density	0.6	2.4 (1.86-3.1)	0.02
Sub-solid Density	1.25	1.82 (1.09-3.03)	< 0.0001
Male Sex	-0.39	3.49 (2.46-4.96)	< 0.0001
Emphysema	-0.03	0.67 (0.53-0.87)	0.85
Multiple Nodules	-0.41	0.97 (0.74-1.28)	< 0.0001

Supplementary Table 3: Coefficients for the clinical variable only model.

Random Effects:			
**Groups**	**Name**	**Variance**	**SD**
Institution	Intercept	5.00	2.24
**Fixed Effects:**			
**Variable**	**Beta**	**OR**	**p**
Age	0.01	1.01 (1-1.02)	0.17
FH	2.05	7.8 (1.48-41.19)	0.02
Smoking	1.15	3.17 (2.03-4.96)	< 0.0001
Size	-0.12	0.89 (0.85-0.93)	< 0.0001
Upper Lobe	0.6	1.81 (1.35-2.44)	< 0.0001
Spiculation	0.94	2.56 (1.75-3.76)	< 0.0001
Contrast	0.58	1.78 (1.32-2.42)	< 0.0001
GGO Density	0.24	1.28 (0.6-2.71)	0.52
Subsolid Density	0.81	2.26 (1.4-3.65)	< 0.0001
Male Sex	-0.3	0.74 (0.55-1)	0.05
Emphysema	-0.06	0.94 (0.68-1.31)	0.72
Multiple Nodules	-0.56	0.57 (0.42-0.78)	< 0.0001
PC1	0.73	2.07 (1.79-2.4)	< 0.0001
PC2	0.2	1.22 (1.07-1.39)	< 0.0001
PC3	-0.4	0.67 (0.5-0.89)	0.01
PC4	-0.73	0.48 (0.38-0.61)	< 0.0001
PC5	-0.27	0.76 (0.4-1.45)	0.41
PC6	-0.24	0.78 (0.54-1.15)	0.21
PC7	-1.13	0.32 (0.19-0.55)	< 0.0001
PC8	-0.6	0.55 (0.3-1.01)	0.06
PC9	-1.12	0.33 (0.19-0.55)	< 0.0001
PC10	-0.46	0.63 (0.39-1.02)	0.06
PC11	-0.83	0.44 (0.14-1.39)	0.16
PC12	-0.74	0.48 (0.31-0.74)	< 0.0001
PC13	-0.23	0.79 (0.35-1.81)	0.58
PC14	0.41	1.51 (0.71-3.19)	0.28
PC15	0.76	2.13 (0.98-4.64)	0.06
PC16	0.3	1.34 (0.85-2.12)	0.20
PC17	-0.4	0.67 (0.32-1.42)	0.30
PC18	-0.32	0.73 (0.4-1.31)	0.29
PC19	-0.19	0.83 (0.43-1.6)	0.57
PC20	0.54	1.72 (0.78-3.81)	0.18

Supplementary Table 4: Coefficients for the combined clinical and radiomics model.

Criteria	Response
Image protocol quality - well-documented image protocols (for example, contrast, slice thickness, energy, etc.) and/or usage of public image protocols allow reproducibility/replicability.	Protocols well documented
Multiple segmentations - possible actions are: segmentation by different physicians/algorithms/software, perturbing segmentations by (random) noise, segmentation at different breathing cycles. Analyse feature robustness to segmentation variabilities.	Yes
Phantom study on all scanners - detect inter-scanner differences and vendor-dependent features. Analyse feature robustness to these sources of variability.	No
Imaging at multiple time points - collect images of individuals at additional time points. Analyse feature robustness to temporal variabilities (for example, organ movement, organ expansion/shrinkage)	Yes
Feature reduction or adjustment for multiple testing - decreases the risk of overfitting. Overfitting is inevitable if the number of features exceeds the number of samples. Consider feature robustness when selecting features	Yes
Multivariable analysis with non radiomics features (for example, EGFR mutation) - is expected to provide a more holistic model. Permits correlating/inferencing between radiomics and non radiomics features	Yes
Detect and discuss biological correlates - demonstration of phenotypic differences (possibly associated with underlying gene–protein expression patterns) deepens understanding of radiomics and biology	No
Cut-off analyses - determine risk groups by either the median, a previously published cut-off or report a continuous risk variable. Reduces the risk of reporting overly optimistic results	Yes
Discrimination statistics - report discrimination statistics (for example, C-statistic, ROC curve, AUC) and their statistical significance (for example, p-values, confidence intervals). One can also apply resampling method (for example, bootstrapping, cross-validation)	Yes
Calibration statistics - report calibration statistics (for example, Calibration-in-the-large/slope, calibration plots) and their statistical significance (for example, P-values, confidence intervals). One can also apply resampling method (for example, bootstrapping, cross-validation)	Yes
Prospective study registered in a trial database - provides the highest level of evidence supporting the clinical validity and usefulness of the radiomics biomarker	No
Validation - the validation is performed without retraining and without adaptation of the cut-off value, provides crucial information with regard to credible clinical performance	Yes
Comparison to 'gold standard' - assess the extent to which the model agrees with/is superior to the current 'gold standard' method (for example, TNM-staging for survival prediction). This comparison shows the added value of radiomics	Yes
Potential clinical utility - report on the current and potential application of the model in a clinical setting (for example, decision curve analysis).	Yes
Cost-effectiveness analysis - report on the cost-effectiveness of the clinical application (for example, QALYs generated)	No
Open science and data - make code and data publicly available. Open science facilitates knowledge transfer and reproducibility of the study	The code is open sourced
**Total Score:**	22 (61%)

Supplementary Table 5: Radiomics Quality Score
